# Advancing the Reproducibility and Repeatability of Capillary Zone Electrophoresis-Mass Spectrometry-Based Top-Down Proteomics by an Improved Capillary Coating Procedure

**DOI:** 10.1021/acs.jproteome.5c01194

**Published:** 2026-03-30

**Authors:** Yifan Yue, Fei Fang, Guangyao Gao, Seyed Amirhossein Sadeghi, Jorge A. Colón Rosado, Reyhane Tabatabaeian Nimavard, Mehrdad Falamarzi Askarani, Lance Thorp, Maryam Rahimzadeh Dashtaki, Guijie Zhu, Liangliang Sun

**Affiliations:** Department of Chemistry, 3078 Michigan State University, East Lansing, Michigan 48824, United States

**Keywords:** capillary zone electrophoresis-mass spectrometry, polyacrylamide coating, top-down proteomics, proteoform, reproducibility

## Abstract

Capillary zone electrophoresis (CZE)-mass spectrometry (MS) has attracted tremendous attention in top-down proteomics (TDP). However, its reproducibility and long-term repeatability for TDP remain concerns, most likely due to capillary coating. Here, we present an improved procedure for making linear polyacrylamide (LPA) coating, the most widely used coating in CE-MS-based proteomics, to boost the reproducibility and long-term repeatability of CZE-MS-based TDP. We focused on the step of degassing the polymerization solution, a critical step for achieving consistent LPA coating quality. The CZE-MS system using LPA-coated capillaries prepared with the optimal degassing procedure produced excellent reproducibility and repeatability for proteoform analysis. The 210 CZE-MS runs of three protein samples (a standard protein mixture, an *E. coli* cell lysate, and a HeLa cell lysate) across 200 h of instrument time with two MS instruments demonstrated the reliability of our conclusion. The optimal condition produced relative standard deviations (RSDs) of less than 2.6% in the migration time of proteoforms across dozens of CZE-MS runs without migration time alignment. CZE-MS/MS analysis of a HeLa whole cell lysate identified 274 ± 20 proteoforms (RSD ∼ 7%) and 146 ± 10 proteins (RSD ∼ 7%) across 10 successive runs and yielded consistent proteoform intensities. The coating procedure can be easily adopted by other research groups, as evidenced by our 2025 CE-MS summer school data. All results demonstrate that CZE-MS using the optimal LPA-coating procedure is ready for broad adoption to enable reproducible and repeatable measurements of proteoforms in complex samples.

## Graphical abstract

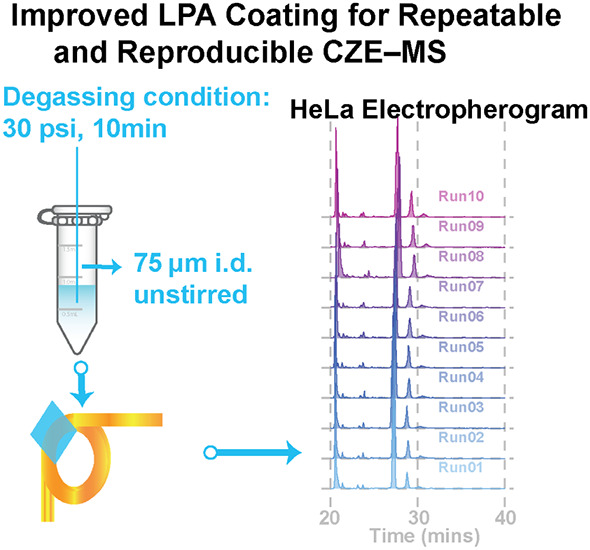

## Introduction

Diseases are usually due to biological processes going awry, which are mainly controlled by proteins. Mass spectrometry (MS)-based top-down proteomics (TDP) offers precise measurement of proteins regarding sequence variations due to gene mutations, alternative RNA splicing, and enzymatic degradation as well as combinations of post-translational modifications (PTMs).^1–7^ “Proteoform” is the common term to describe all these different forms of protein molecules produced from the same gene.^8^ MS-based TDP measurement of proteoforms in complex biological systems provides unprecedented opportunities for advancing fundamental and translational research, for example, the discovery of novel disease biomarkers for more effective diagnosis and therapeutic development.^9–12^

TDP is increasingly recognized as a powerful tool for studying disease-related proteoform alterations. Alzheimer’s disease, characterized by β-amyloid deposition, tau pathology, oxidative stress, and inflammation, has been investigated using TDP.^13^ TDP has also been applied to therapeutic antibodies, which are widely used to treat cancer, autoimmune disorders, and infectious diseases, with Khristenko et al. outlining strategies for characterizing emerging monoclonal antibody modalities.^14^ Olianas et al. used a salivary TDP workflow to differentiate two autoimmune liver diseases—autoimmune hepatitis, marked by immune-driven hepatic inflammation, and primary biliary cholangitis, involving immune-mediated small-duct destruction.^15^ Cardiovascular disease, a broader category encompassing all disorders of the heart and vasculature, together with cancer and neurodegenerative diseases, has been widely reviewed as an important application area for TDP.^4,10,16–18^

MS-based TDP still faces challenges due to the extremely high complexity of proteoforms in cells, tissues, and biological fluids.^19^ The analytical challenge requires high-resolution separations of proteoforms before MS analysis. Our research group and others have demonstrated the capability of capillary zone electrophoresis (CZE)-MS for high-resolution separation and highly sensitive detection of proteoforms in various complex biosystems, e.g., bacteria,^20–23^ yeast,^24–27^ human cancer cells,^28–31^ brain tissue,^32–37^ biological fluids,^38–41^ and biopharmaceuticals.^42–47^ With the advanced mass spectrometers, CZE-MS achieved the identification of thousands or even over ten thousand proteoforms in complex samples^21,28,33,48^ and enabled the measurement of proteoforms in mass-limited samples (i.e., single or few cells).^30,31,35^ However, the long-term repeatability and reproducibility of CZE-MS-based TDP are still a concern, most likely due to the inner wall coating of the CZE separation capillary, although some recent studies have tried to solve this issue. Sadeghi et al. employed periodical cleaning of the inner surface of the capillary coated by linear polyacrylamide (LPA) using diluted ammonium hydroxide and achieved consistent proteoform separations and identifications across over 20 runs after hours of stabilization and equilibration of the capillary inner wall.^25^ Wang et al. developed a cationic capillary coating to improve the separation and reproducibility of CZE-MS for proteoforms.^24^

Based on our 10 years of experience with LPA-coated capillaries for CZE-MS-based proteomics,^3,12,49,50^ we further improved the LPA coating procedure focusing on the ease of repeatable and reproducible preparation of LPA-coated capillaries for broad adoption of CZE-MS-based TDP. However, the quality of LPA-coated capillaries can vary greatly from person to person, which is most likely due to slight variations in one critical step of the procedure, namely the degassing of the polymerization solution containing acrylamide (monomer) and ammonium persulfate (APS, initiator) using nitrogen to remove oxygen from the solution for an efficient polymerization reaction. Oxygen inhibits free radical polymerization, so thorough degassing is crucial for a uniform polyacrylamide layer. Here, we systematically investigated the degassing procedure to improve the consistency and reproducibility of LPA-coated capillaries. We evaluated the separation performance of LPA-coated capillaries prepared under four different degassing conditions using a standard protein mixture, *E. coli* lysate, and HeLa cell lysate across a total of 210 runs, which required over 200 h of operation. More importantly, the optimal procedure was tested by our 2025 CE-MS summer school participants to investigate the ease of broad adoption.

## Experimental Section

### Materials

All reagents were obtained from Sigma-Aldrich (St. Louis, MO, USA) unless stated otherwise. LC-MS grade solvents, including water, methanol, formic acid, and acetic acid were purchased from Fisher Scientific (Pittsburgh, PA, USA). Acrylamide was purchased from Acros Organics (NJ, USA), and fused silica capillaries (50 μm i.d./360 μm o.d.) were ordered from Polymicro Technologies (Phoenix, AZ, USA).

### Capillary Pretreatment

Fused silica capillaries (50 μm i.d./360 μm o.d.) were pretreated using a syringe pump with 1 M hydrochloric acid, water, 1 M sodium hydroxide, water, and finally methanol following our typical procedure.^38,49,51,52^ Next, the pretreated capillaries were dried with nitrogen gas (30 psi) at room temperature for 4 h. Subsequently, 50% (v/v) 3-(trimethoxysilyl) propyl methacrylate in methanol was flushed through each capillary, ensuring the entire capillary was filled. Both ends of the capillaries were then sealed with silicone rubber, and the capillaries were incubated at room temperature for approximately 2 days. After incubation, each capillary was rinsed with methanol and dried under nitrogen for an additional 2 h. After that, both ends of the capillaries were sealed with silicone rubber for storage at room temperature before use.

### Degas the Polymerization Solution

The acrylamide solution was prepared by dissolving 40 mg of acrylamide in 1 mL of deionized water. The APS solution was prepared by dissolving 5 mg of APS in 100 μL of deionized water. For the preparation of each LPA-coated capillary, 500 μL of the acrylamide solution was mixed with 3.5 μL of the APS solution in a 1.5 mL Eppendorf tube, followed by vortexing. A small hole was created in the cap of the Eppendorf tube to insert a capillary for degassing, with the hole providing a tight fit. The acrylamide and APS mixture was degassed under four conditions by bubbling nitrogen gas (30 psi) through the 15 cm capillaries (50 or 75 μm i.d.) for 10 min, as illustrated in Figure 1A. Two LPA-coated capillaries were prepared for each condition described below: one was used for the standard protein mixture, and the other for the *E. coli* sample. After identifying the optimal degassing condition, two additional capillaries were prepared under this degassing condition by two different lab members to assess reproducibility.

**Figure 1:**
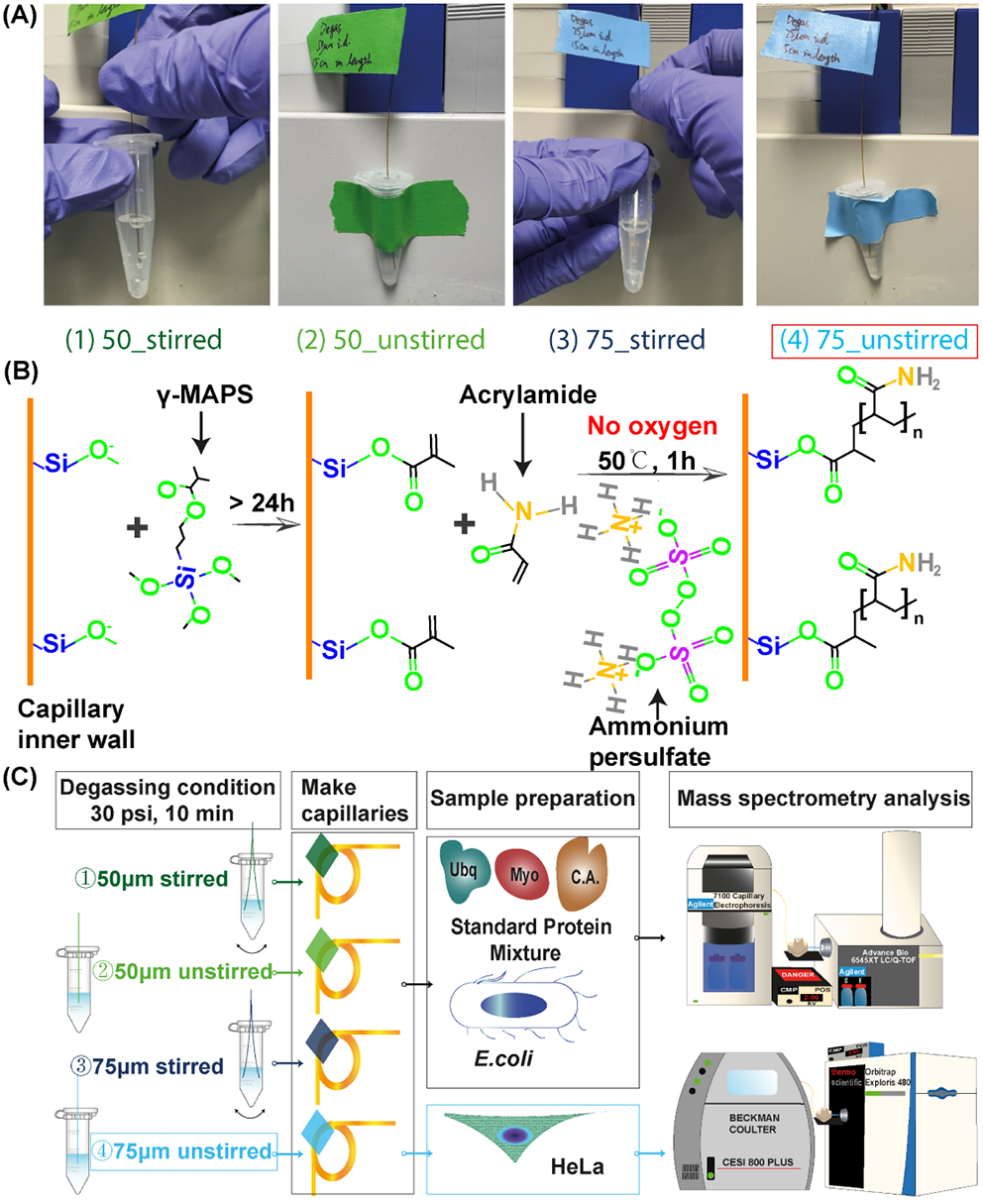
(A) Representative images of the four degassing conditions: (1) 50_stirred—large bubbles observed during degassing; (2) 50_unstirred—very small bubbles; (3) 75_stirred—large bubbles; (4) 75_unstirred—small and continuous bubbles. (B) LPA-coating process. The ammonium persulfate (APS) initiated polymerization reaction requires no oxygen environment, suggesting the importance of degassing the polymerization solution. (C) Schematic overview of the entire experiment.

*Condition 1 (* 50_stirred): A 50 μm i.d. capillary was used to degas the solution while manually stirring with the same capillary.

*Condition 2 (*50_unstirred): A 50 μm i.d. capillary was used to degas the solution without stirring. The 1.5 mL Eppendorf tube was fixed vertically on the wall and remained stationary during degassing. The end of the capillary was kept close to the middle in the solution in the vertical direction as shown in Figure 1A.

*Condition 3 (*75_stirred): A 75 μm i.d. capillary was used to degas the solution with manual stirring.

*Condition 4 (*75_unstirred): A 75 μm i.d. capillary was used to degas the solution without stirring, with the Eppendorf tube fixed and kept stationary as described in *Condition 2*.

### Preparation of LPA-Coated Capillaries

After degassing, each coating solution was introduced into a 100 cm-long, 50 μm i.d. pretreated capillary by vacuum. Both ends of each capillary were sealed with silicone rubber, and the capillaries were incubated in a 50 °C water bath for 1 h. Subsequently, the capillaries were flushed individually with water to remove excess reagents. The distal ∼1 cm of each capillary tip was etched with hydrofluoric acid to reduce the outer diameter to approximately 70 μm, while preserving the inner diameter, as described in ref 53. *Caution: After etching with hydrofluoric acid, the etched part of the LPA-coated capillaries needs to be washed with water thoroughly before any further experiments. Please follow the safety procedures when handling hydrofluoric acid.*

### Sample Preparation

A standard protein solution was prepared by dissolving four proteins in 200 μL of 5% (v/v) acetic acid in water. The solution was aliquoted into ten microcentrifuge tubes (20 μL per tube) and stored at −20 °C until use. The final concentrations of each protein were as follows: myoglobin, 0.2 mg/mL; bovine serum albumin (BSA), 0.7 mg/mL; carbonic anhydrase, 0.3 mg/mL; and ubiquitin, 1 mg/mL.

*E. coli* cells (strain MG1655) were cultured based on our published work.^20^ The *E. coli* cell (strain MG1655) pellet (0.35 g) was dissolved in 2 mL of the extraction buffer (8 M urea, 100 mM ammonium bicarbonate (ABC), complete protease inhibitor). The sample was then sonicated for 2 min twice in an ice bath with a Branson Sonifier 250 (VWR Scientific). After that, the cell lysate was centrifuged at 14000 × *g* for 25 min. The concentration of the collected supernatant was determined using a bicinchoninic acid (BCA) kit. The *E. coli* cell lysate sample was buffer-exchanged using 10 kDa cutoff Amicon Ultra centrifugal filters. To prepare the filters, 250 μL of 100 mM ABC was added to each filter, followed by centrifugation at 14,000 × *g* for 20 min at 15 °C to prewet the membranes. Subsequently, 67 μL of the *E. coli* lysate (about 200 μg of proteins) was loaded into each prewetted filter. Then, 133 μL of 100 mM ABC was added to each filter and centrifuged at 14,000 × *g* for 20 min at 15 °C. The filtrate collected at the bottom was carefully discarded. Next, 200 μL of 100 mM ABC was added to each filter, followed by centrifugation at 14,000 × *g* for 15 min at 15 °C. This step was repeated three times. Finally, the retained material from filters was collected for subsequent experiments. The *E. coli* sample was diluted to about 1 mg/mL using 100 mM ABC for CZE-Q-TOF analysis.

HeLa cells were cultured in MEM-containing 10% fetal bovine serum (v/v) and maintained in a humidified atmosphere of 95% air and 5% CO_2_ at 37 °C. The adherent cell layer was washed with PBS and then trypsinized with 0.05% trypsin-EDTA solution for 5 min at 37 °C. Then, the cells were centrifuged at 250 × *g* for 5 min to remove trypsin, followed by washing with PBS three times. HeLa cell proteins were extracted using Dulbecco’s phosphate-buffered saline (DPBS, Sigma-Aldrich, MO, USA). Three mL of DPBS containing 1% protease and phosphatase inhibitors was used. The sample was divided equally into two 1.5 mL vials to generate technical duplicates. After the cell pellet was resuspended in DPBS, the resulting duplicates were lysed on ice for 20 min by sonication with a Branson Sonifier 250 (VWR Scientific, IL, USA) at 15 s on/off intervals. The lysate was then centrifuged with an Allegra X-30R Centrifuge (Beckman Coulter, CA, USA) at 14,000 × *g* for 30 min at 4 °C, and the supernatant containing the extracted proteins was collected. Duplicates were combined and protein concentration was determined to be 3.3 mg/mL using a bicinchoninic acid (BCA) assay (Thermo Fisher Scientific, MA, USA).

The HeLa cell lysate sample was buffer-exchanged using 100 kDa and 10 kDa cutoff Amicon Ultra centrifugal filters. To prepare the filters, 250 μL of 100 mM ABC was added to each filter, followed by centrifugation at 14,000 × *g* for 15 min at 10 °C to prewet the membranes. Subsequently, 98 μL of HeLa lysate (about 324 μg of proteins) was loaded into each of the prewetted 100 kDa filters. Then, 102 μL of 100 mM ABC was added to each filter and centrifuged at 14,000 × *g* for 15 min at 10 °C. The filtrate collected from each 100 kDa filter was then transferred to the corresponding 10 kDa filters.

Following sample transfer, 200 μL of 100 mM ABC was added to each 10 kDa filter and centrifuged at 14,000 × *g* for 15 min at 10 °C. This step was repeated three times. Finally, the retained material from the filters was collected for subsequent experiments. The HeLa lysate sample was diluted to about 1 mg/mL using 100 mM ABC for CZE-MS analysis.

### CZE-Q-TOF for the Standard Protein Mixture and *E. coli*Cell Lysate

The CZE experiments were performed using a 7100 CE System from Agilent Technologies (Santa Clara, CA). The CZE system was coupled to a 6545XT AdvanceBio Q-TOF mass spectrometer (Agilent Technologies, Santa Clara, CA) via an electrokinetically pumped sheath-flow nanospray interface (EMASS-II CE-MS Ion Source, CMP Scientific, Brooklyn, NY).^54,55^ The sheath buffer consisted of 10% (v/v) methanol and 0.2% (v/v) formic acid in water. The electrospray ionization (ESI) emitters for the CZE-MS interface were made by pulling borosilicate glass capillaries (1.0 mm outer diameter, 0.75 mm inner diameter, 10 cm in length) using a Sutter P-1000 flaming/brown micropipette puller. The final emitter opening size was 30–40 μm. The applied ESI voltage ranged between +2.0 kV and +2.2 kV, and the distance between the emitter tip and the ion transfer tube was about 4 mm.

CZE separations were conducted under standard conditions employing a 1 m-long capillary with a separation voltage of 30 kV. A 5% (v/v) acetic acid solution (pH 2.4) was used as the background electrolyte (BGE). The sample injection volume was approximately 60 nL per run for the standard protein mixture and 50 nL per run for the *E. coli* cell lysate. The separation time was 40 min per run for the standard protein mixture and 60 min per run for the *E. coli* cell lysate. Following each run, the capillary was flushed and cleaned with BGE for 10 min at 950-mbar pressure.

For MS parameters, the gas temperature was 320 °C, the flow rate of the nitrogen drying gas was 1 L/min, the voltage applied to the ion transfer tube was 0 V, the range of data acquisition was 600–2500 *m*/*z*, and the acquisition rate was 0.4 spectra per second.

### CZE-MS/MS for Analyzing a HeLa Cell Lysate

A Beckman CESI 8000 capillary electrophoresis autosampler (Sciex) was connected to an Orbitrap Exploris 480 mass spectrometer (Thermo Fisher Scientific) via an electro-kinetically pumped sheath flow CE-MS interface (CMP Scientific, Brooklyn, NY).^54,55^ The spray voltage was approximately +2 kV, with a 2 mm distance between the emitter tip and the ion transfer tube. For CZE separation, the 1 m-long capillary prepared under the optimized degassing condition was used. The BGE and sheath buffer were the same as those used for the *E. coli* sample. The sample injection volume was approximately 36 nL (∼60 ng protein material), achieved with a 10-s injection at 5 psi. The separation voltage was set at 30 kV, with a separation time of 75 min. After each run, the capillary was cleaned with BGE for 15 min at 20 psi pressure, 30 kV voltage.

The Orbitrap Exploris 480 mass spectrometer in Data-Dependent Acquisition (DDA) mode was employed. The full MS parameters included a high mass resolution of 480,000 (at *m*/*z* 200) with a single microscan, covering a scan range of 600–2000 *m*/*z*. Precursor ions were isolated with a 2 *m*/*z* window and subjected to fragmentation through higher-energy collisional dissociation (HCD) with a normalized collision energy of 25%. Only precursor ions with an intensity exceeding 5E4 and a charge state ranging from 3 to 60 were selected for fragmentation. Product ions were detected with a resolution of 60,000 (at 200 *m*/*z*), utilizing a single microscan, and maintaining a normalized AGC target value of 300% for both conditions. Dynamic exclusion was enabled with a duration of 15 s. The precursor mass tolerance was 10 ppm. Additionally, the “Exclude isotopes” function was activated.

### Proteoform Identification and Data Analysis

HeLa cell proteoform identification and quantification were performed using the TopPIC software (version 1.7.8).^56^ The RAW files were first converted to mzML format through MSConvert from ProteoWizard.^57^ Spectral deconvolution was performed with TopFD,^58^ integrated within the TopPIC package (version 1.7.8), using default parameters. Database searches were conducted using the TopPIC feature in the package against the UniProt *Homo sapiens* proteome database (UP000005640, 83587 entries, accessed on 04/07/2025). The mass error tolerance was set to 10 ppm, with a maximum mass shift of 500 Da for unknown modifications. False discovery rate cutoffs were set at 1% for proteoform-spectrum matches (PrSMs) and 5% for proteoforms.

Data processing and visualization were performed using R version 4.4.1. Base R packages were used along with the following additional packages: ggplot2,^59^ ggdist,^60^ ggbreak,^61^ and patchwork^62^ for data visualization; readxl,^63^ dplyr,^64^ and tidyr^65^ for data cleaning and wrangling; and ggpubr^66^ for performing Student’s *t* tests. All the scripts for data analysis and data visualization are provided as open source https://github.com/YiFanYUE99/Degas_experiment/.

## Results and Discussion

CZE-MS-based TDP usually employs neutral and hydrophilic LPA-coated capillaries to reduce nonspecific proteoform adsorption to improve the separation window of proteoforms and allow acquisition of more MS/MS spectra. Our group has been utilizing the LPA-coated capillaries in CZE-MS-based TDP for nearly 10 years, and the LPA-coating was prepared based on the procedure developed by Zhu et al.^49^ with some modifications.^38,51,52^ The thermally initiated free radical polymerization process formed linear polymers of acrylamide on the capillary inner wall via the reactions with the C=C bond, and the process requires an oxygen-free environment to maximize the performance (Figure 1B). In this work, we investigated four different degassing conditions of the solution to determine the optimal approach for reproducible preparation of LPA-coated capillaries. We investigated the long-term reproducibility and repeatability of CZE-MS-based TDP of various proteoform mixtures with two main types of CE systems and mass spectrometers (TOF and Orbitrap) (Figure 1C).

### Evaluation of LPA-Coated Capillaries from Different Degassing Approaches by CZE-MS Analysis of a Standard Protein Mixture

A standard protein mixture containing bovine serum albumin (BSA), myoglobin (myo), carbonic anhydrase (CA), and ubiquitin (ubi) was used to evaluate the LPA-coated capillaries for CZE-MS on a Q-TOF instrument. Twenty CZE-MS runs were performed on each of the four capillaries coated under the four degassing conditions shown in Figure 1A (80 runs in total). The *50_unstirred* capillary failed after 18 runs and exhibited the highest full width at half-maximum (fwhm) of protein peaks and the lowest number of theoretical plates during its usable lifespan. Therefore, we excluded this degassing condition from subsequent data analysis for the standard protein mixture. Of all tested conditions, the 75_unstirred condition demonstrated the most consistent performance, evidenced by the lowest relative standard deviations (RSDs) in migration time, number of theoretical plates, and separation resolution across the 20 CZE-MS runs, Table 1. Figure S1A shows an example electropherogram of the standard protein mixture with well-resolved protein peaks. Figure S1B–S1E show the electropherograms of all the 80 CZE-MS runs.

**Table 1:** Summary of Migration Time (MT, Min), Number of Theoretical Plates (N), and Separation Resolution (R) of the Standard Protein Mixture^a^

Capillary	MT (Mean/RSD) Ubi	MT (Mean/RSD) Myo	MT (Mean/RSD) CA	N (Mean/RSD) Ubi	R (Mean/RSD) Ubi vs Myo
50_stirred	17.1/2.7%	18.5/2.9%	20.2/2.8%	13609/15% ns	2.1/10.7%**
75_stirred	17.2/3.5%	18.6/3.6%	20.4/3.9%	9652/11%****	1.8/8.2%*
75_unstirred	14.9/2.4%	16.0/2.5%	17.4/2.6%	12964/9%	1.9/6.7%

a The abbreviations Ubi, Myo, and CA refer to Ubiquitin, Myoglobin, and Carbonic Anhydrase, respectively. Student’s *t* test was conducted to compare the 75_unstirred condition with the other conditions, and significance levels are indicated by asterisks. (**** *p* ≤ 0.0001; *** *p* ≤ 0.001; ** *p* ≤ 0.01; * *p* ≤ 0.05; ns, not statistically significant).

As illustrated in Figure 2, the 75_unstirred condition yielded the most favorable results among all tested conditions, including a reduced migration time range and lower fwhm values across all three protein peaks.

**Figure 2:**
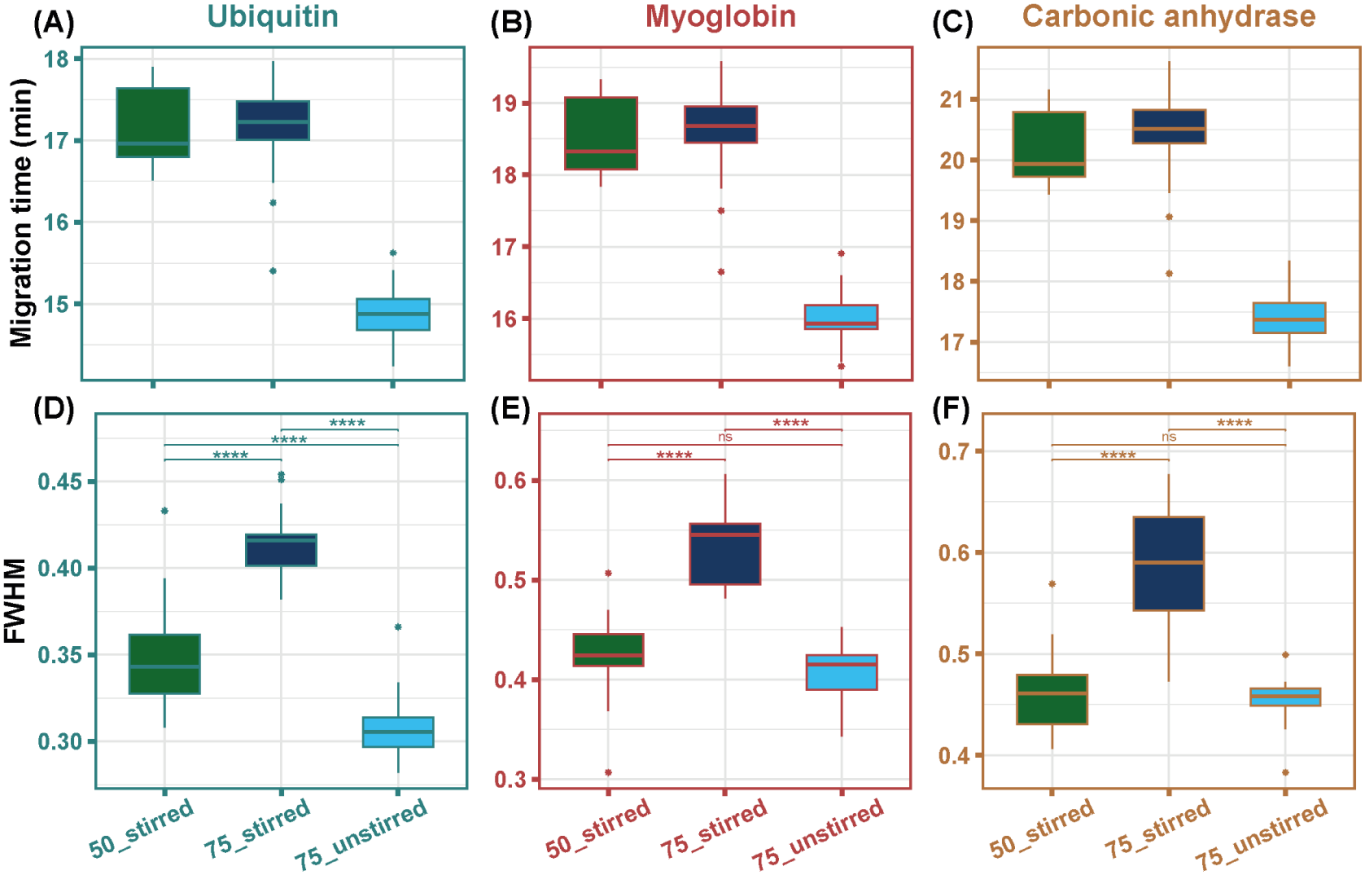
Boxplots of migration time (A, B, C) and fwhm (D, E, F) across the three degassing conditions. Statistical significance was determined using Student’s *t* test. The significance levels are indicated by asterisks. (**** *p* ≤ 0.0001; *** *p* ≤ 0.001; ** *p* ≤ 0.01; * *p* ≤ 0.05; ns, not statistically significant).

### Evaluation of the LPA Capillary Coating by CZE-MS Analysis of an *E. coli*Cell Lysate

Approximately 1 mg/mL of an *E. coli* lysate was analyzed by CZE-Q-TOF using four different capillaries, each coated under distinct degassing conditions. A total of 80 runs were performed—20 runs per capillary. Several peak characteristics—including migration time RSD, intensity, number of theoretical plates, and resolution—were evaluated to determine the optimal degassing condition. Figure 3 shows a representative base peak electropherogram of the *E. coli* sample. Given the complexity of the *E. coli* lysate, we focused on two well-resolved, most abundant proteoform peaks (labeled Peak 1 and Peak 2 in Figure 3) as representative indicators for comparing separation performance across coating conditions. Peak 1 and Peak 2 were selected for analysis because of their high abundance, which made the comparisons more reliable. Peak 1 was extracted at *m*/*z* 1083.03666 ± 0.05, and Peak 2 at *m*/*z* 1331.49046 ± 0.05, from all MS raw files to obtain data for comparison. The 5–10 min region was selected as the noise baseline for subsequent signal-to-noise ratio (S/N) analysis.

**Figure 3:**
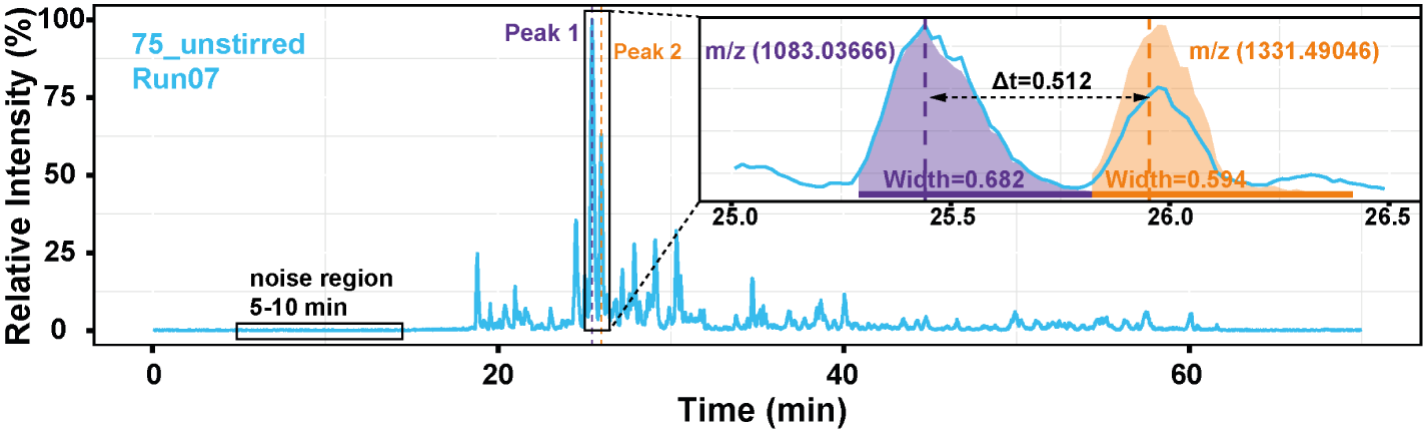
Electropherogram of the *E. coli* sample from Run 7 of a 75_unstirred capillary. Peak 1 is a protein of about 9.8 kDa, and Peak 2 is a protein of about 12 kDa.

Based on the mass spectra shown in Figure 4A and B, Peak 1 (purple) corresponds to a protein with an approximate molecular weight of 9.8 kDa, while Peak 2 (orange) corresponds to a protein of approximately 12 kDa. In Figure 4C and D, the 75_unstirred condition consistently exhibits the smallest migration time interval, whereas the 50_unstirred condition displays a pronounced tendency toward peak shifting. Figure 4E and F demonstrate that the 75_unstirred condition exhibits significantly higher signal intensity compared to both the 50_stirred and 50_unstirred conditions, and a noticeably—but not statistically significant—increase in intensity compared to the 75_stirred condition. Figure 4G and H show that the number of theoretical plates is higher under the 75_unstirred condition, regardless of whether the calculation is based on Peak 1 or Peak 2, or whether fwhm or peak width is used.

**Figure 4:**
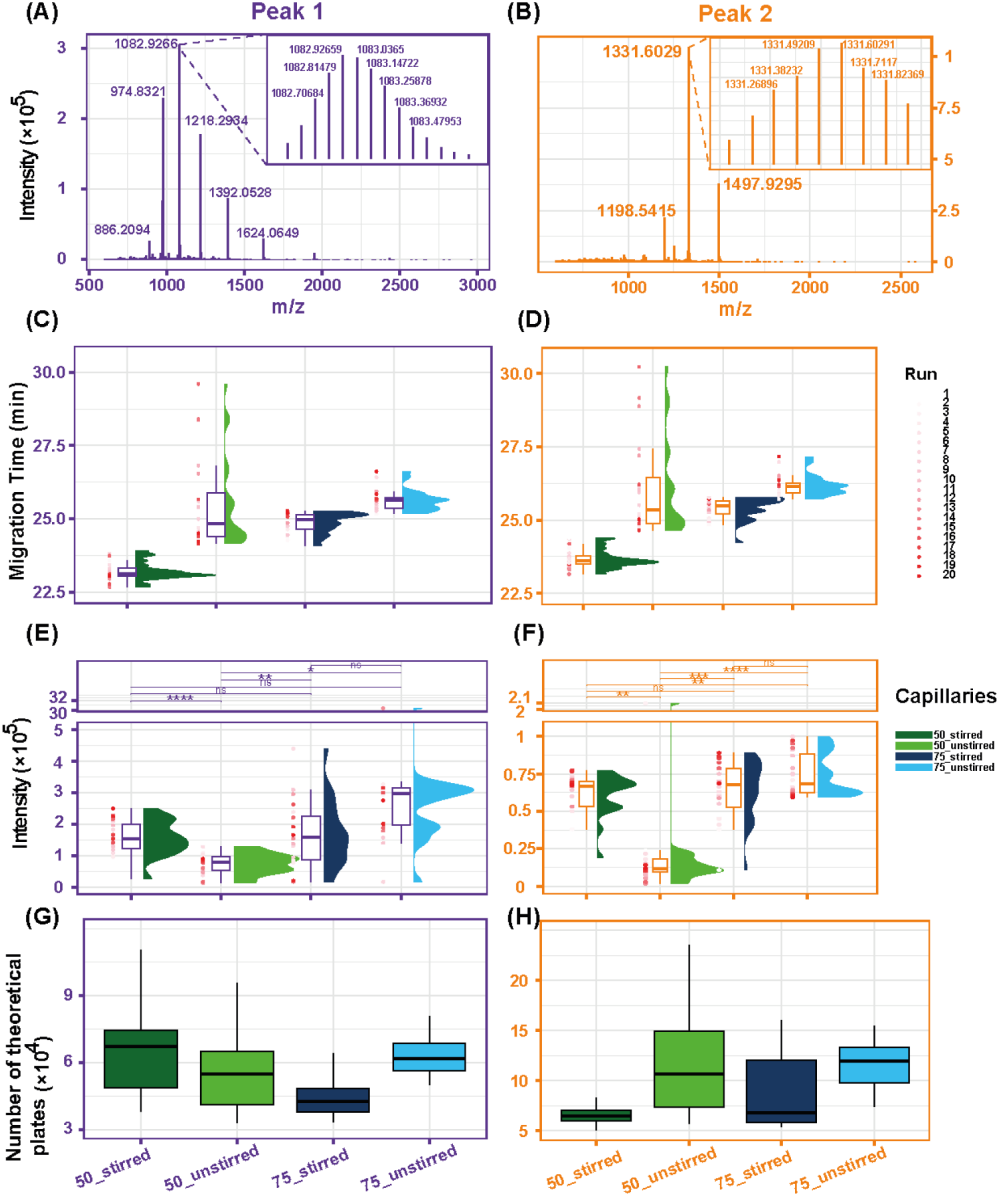
Mass spectra of Peak 1 and Peak 2 (A, B) in Figure 3. Boxplots of migration time (C, D), proteoform intensity (E, F), and the number of theoretical plates (G, H) across the four degassing conditions. For each degassing condition, 20 CZE-MS runs were performed. Figures A, C, E, and G are for Peak 1. Figures B, D, F, and H are for Peak 2. Significance levels follow the same notation as in Figure 2.

In Figure S2 and Table S1, the signal-to-noise ratios (SNRs) of Peak 1 and Peak 2 under the 75_unstirred condition were stable and higher than those observed under 50_unstirred and 50_stirred. Peak 1 showed an SNR of 471 (RSD = 65.5%), and Peak 2 had 208 (RSD = 27.2%), both significantly higher than the 50_unstirred condition (*p* < 0.001). The separation performance of the 75_unstirred capillary is consistent with close-to-baseline separations. In contrast, the 50_unstirred condition demonstrated the poorest performance, with greater migration time variability and more peak overlap.

Then, we summarized the migration time, the number of theoretical plates, and separation resolution data in Table S2. The 75_unstirred capillary produced the smallest migration time variations, the highest number of theoretical plates, and the best separation resolution between Peak 1 and Peak 2 compared to the other three conditions. The 75_unstirred condition was determined to be the optimal degassing condition for LPA-coating preparation and was used in all further experiments.

### Reproducibility of LPA Coating Preparation

Based on the results above, we concluded that 75_unstirred represents the optimal degassing condition. Possible reasons include: (1) the 50 μm i.d. capillary may not generate sufficient nitrogen flow, particularly under the 50_unstirred condition; and (2) the stirring process in the 75_stirred condition may enlarge the hole in the tube cap, allowing air (oxygen) to leak into the tube.

To further evaluate the reproducibility of capillary preparation under this optimal condition, two additional capillaries were fabricated by two different individuals and used to analyze the *E. coli* sample, with 20 CZE-MS runs performed on each. The newly prepared capillaries were designated as 75_unstirred_2 and 75_unstirred_ZHU, where 75_unstirred_2 was prepared by the same individual who made 75_unstirred_1, and 75_unstirred_ZHU was prepared by a different person. The previously tested capillary was renamed 75_unstirred_1 for clarity. Detailed performance metrics (migration time, intensity, and separation resolution) of the three capillaries are shown in Figure S3 and Table S3. Each LPA-coated capillary produced consistent migration time and proteoform intensities as well as separation resolution between Peak 1 and Peak 2 across the 20 CZE-MS runs. Notably, although the capillaries were prepared under identical degassing conditions, variations in migration time of Peak 1 (Figure S3A) and Peak 2 (Figure S3B) were clearly observed, likely due to slight differences in the details of personal operations and CE-MS setup among individuals. We note that the three LPA-coated capillaries yielded similar RSDs in migration time and comparable separation resolution between Peak 1 and Peak 2 from the 20 runs (Table S3). The migration time differences between capillaries can be easily corrected by migration time alignment if needed in real large-scale TDP applications. The three LPA-coated capillaries generated reasonably consistent intensity of proteoforms in Peak 1 and Peak 2 with a less than 2-fold difference in the median intensity from the 20 CZE-MS runs (Figure S4C and S4D).

The Sun lab has organized two CE-MS summer schools in 2024 and 2025. In the 2025 summer school, we had nine graduate students/postdocs/research scientists from 7 research groups. Those participants had substantially different levels of knowledge and experience in CZE-MS-based TDP. They followed our optimized degassing procedure and made five LPA-coated capillaries for CZE-MS analysis of a standard protein mixture. The five LPA-coated capillaries they made produced consistent separation profiles of the standard protein mixture, except for the “Capillary_1” (Figure S4). For “Capillary_1”, there were some mistakes during the coating preparation, and we expected significantly lower separation efficiency of “Capillary_1” (much wider protein peaks) compared to other capillaries. The data clearly demonstrates the high potential of the LPA-coating procedure for broad adoption. In the end, one of the LPA-coated capillaries was used to perform an overnight analysis of the *E. coli* cell lysate, producing high-capacity separation of the complex proteoform mixture with excellent repeatability across nine successive runs, Figure S5, indicating the high quality of the LPA-coating for TDP analysis of complex proteomes. The CE-MS summer school data provided us with enough confidence that our CZE-MS technique is ready for broad adoption to enable large-scale TDP of complex biological samples.

### Applying CZE-MS/MS with the New LPA Coating Procedure for TDP of HeLa Cells

To validate the performance of CZE-MS/MS with the improved LPA-coating for TDP analysis of a biological sample with extremely high complexity, we performed CZE-MS/MS analysis of a HeLa cell lysate for 10 technical replicates. The CZE-MS/MS runs produced consistent separation profiles and a normalized proteoform intensity level of 3.48 × 10^8^ ± 9.29% (Mean ± RSD) across the 10 runs (Figure 5A and Figure S6). In Figure S6, the HeLa electropherogram exhibits a highly stable separation profile with strong run-to-run reproducibility, underscoring the robustness of the capillary-coating procedure. CZE-MS/MS also generated relatively consistent numbers of proteoform and protein identifications (Figure 5B). The RSDs of the number of proteoform and protein identifications are smaller than 7.6% and 7.0%. As shown in Figure 5C, the proteoform overlap between any pairs of CZE-MS/MS runs ranged from 33% to 44%, and the pairwise protein overlap ranged from 53% to 72%, indicating that CZE-MS/MS had reasonable consistency in identifying the same proteoforms from an extremely complex biological sample considering the randomness of the data-dependent acquisition (DDA) approach used here. Twenty-eight proteoforms were selected to study the proteoform intensity consistency across the 10 CZE-MS/MS runs, as shown in Figure 5D. The data suggests high consistency of proteoform intensity across these 10 runs. The HeLa cell data fully demonstrates the capability of CZE-MS/MS, with the improved LPA-coating procedure, for reproducible measurement of proteoforms in extremely complex biological samples. The proteoforms and proteins identified in this study are listed in Supporting Information II.

**Figure 5:**
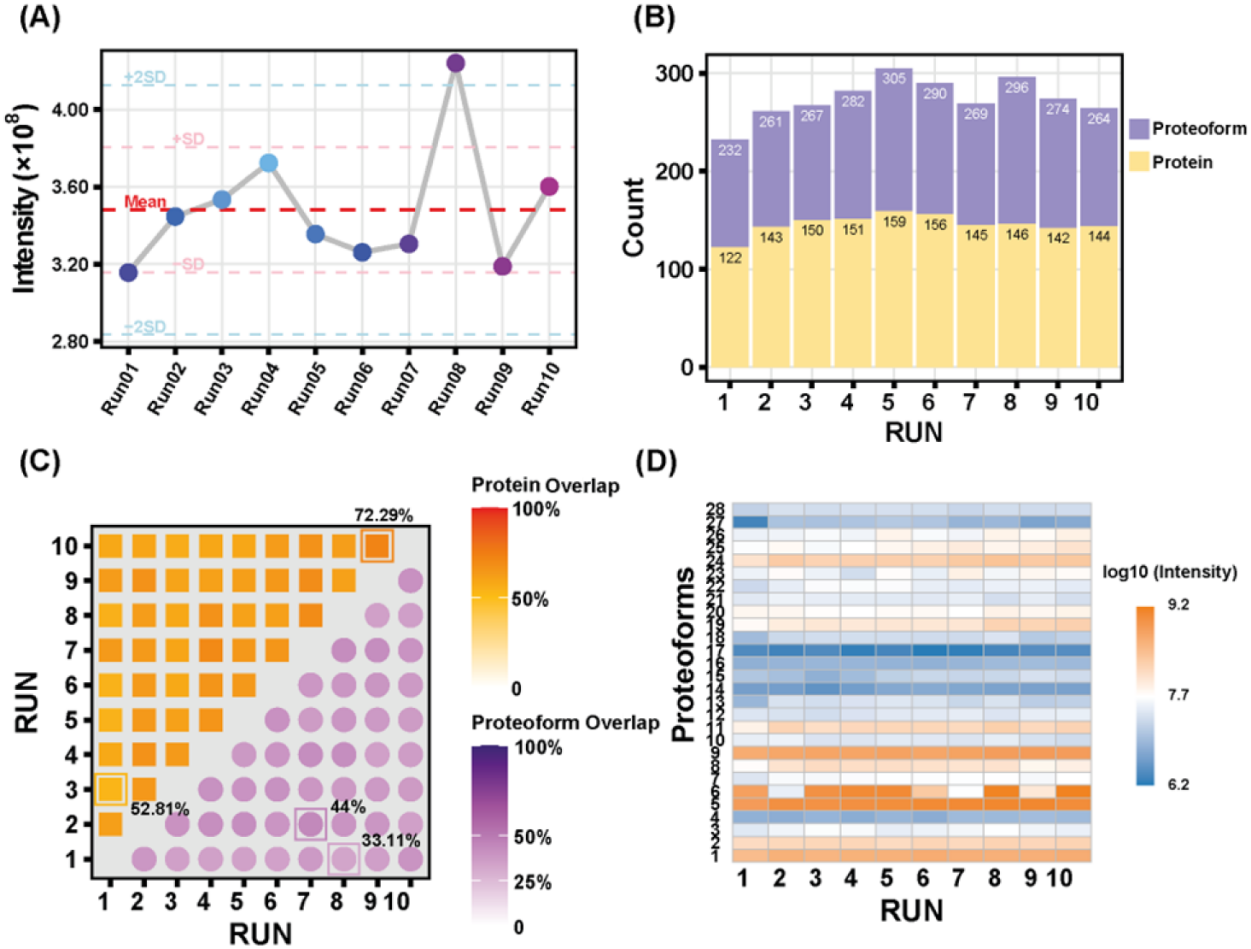
Summary of the HeLa cell lysate data. (A) Normalized proteoform intensity levels across 10 HeLa cell runs. (B) The number of protein and proteoform identifications across the ten CZE-MS/MS runs. (C) Protein (yellow) and proteoform (purple) overlaps between any two CZE-MS/MS runs. The overlap percentage was calculated as the intersection over union of identified proteoform/protein sets and then multiplied by 100%. (D) Heatmap of intensities of 28 shared proteoforms across the 10 CZE-MS/MS runs. The proteoform intensity was obtained from the TopPIC database search. The proteoform indices correspond to the proteoforms listed in Supporting Information II, sheet: Shared_Proteoform.

## Conclusions

We systematically studied the degassing procedure to achieve better reproducibility and repeatability of CZE-MS for TDP applications. Based on a large-scale evaluation of 210 CZE-MS runs on samples ranging from standard protein mixtures to *E. coli* and HeLa cell lysates, we conclude that the optimal degassing condition is using a 75 μm i.d. capillary (15 cm in length) for degassing under 30 psi pressure for 10 min without stirring. The CZE-MS system using the LPA-coated capillaries prepared under the optimal condition produced consistent separation profiles, separation efficiency, and proteoform intensity across dozens of runs for complex proteomes. The LPA-coated capillaries prepared by different individuals produced reasonably consistent CZE-MS separation and detection performance, indicating that this improved method can be readily adopted by other laboratories to enhance TDP workflows. Moreover, it paves the way for more reliable large-cohort TDP studies, where reproducibility is paramount.

Because the degassing performance for removing oxygen is vital for the successful preparation of the LPA coating, the real-time monitoring of the degassing process regarding oxygen concentration in the polymerization solution will provide accurate control and a better understanding of the reaction. We will undertake a new study focusing on this purpose. In addition, we will continue improving the LPA coating procedure by investigating, e.g., a hermetically sealed stirring apparatus, to further enhance degassing performance potentially.

Because oxygen inhibition is a general concern in free-radical polymerization, the degassing strategy described here may also be beneficial for other capillary coatings prepared via similar mechanisms, such as polydimethylacrylamide (PDMA) and [poly­(acrylamide-*co*-(3-acrylamidopropyl)­trimethylammonium chloride) (PAMAPTAC)].^67,68^

## Supplementary Material





## Data Availability

The CZE-MS/MS data of the HeLa cells have been deposited to the ProteomeXchange Consortium via the PRIDE partner^69^ repository with the data set identifier PXD067278.

## Notes

Signal-to-noise ratio and relative standard deviation for peaks P1 and P2 under different capillary and stirring conditions (Table S1); migration time, the number of theoretical plates, and separation resolution of Peaks 1 and 2 (Table S2); summary of migration time and separation resolution of the three capillaries made under the same degassing conditions (Table S3); electropherograms and mass spectra of the standard protein mixture obtained using LPA-coated capillaries prepared with different degassing procedures (Figure S1); comparison of peak separation and signal-to-noise ratios for two proteins using LPA-coated capillaries prepared under different degassing conditions (Figure S2); migration time and intensity boxplots of two proteins from the *E. coli* sample obtained using LPA-coated capillaries prepared by two individuals under the same degassing condition (Figure S3); electropherograms of a standard protein mixture obtained using capillaries prepared by five individuals during the 2025 CE-MS summer school (Figure S4); electropherograms of *E. coli* cell lysate obtained using an LPA-coated capillary prepared by 2025 CE-MS summer school participants following the optimized degassing procedure (Figure S5); electropherograms of HeLa cell lysate obtained using an LPA-coated capillary prepared under the 75_unstirred condition (Figure S6) (PDF)
List of proteoforms identified from a HeLa cell lysate (XLSX)
