# Supplementary material for: Advancing the Reproducibility and Repeatability of Capillary Zone Electrophoresis-Mass Spectrometry-Based Top-Down Proteomics by an Improved Capillary Coating Procedure
Source: J Proteome Res. 2026 Mar 30;25(5):2452–61. doi: 10.1021/acs.jproteome.5c01194 (PMC13140149; doi:10.1021/acs.jproteome.5c01194)
Supplement: Supplementary file 1 [file pr5c01194_si_001.pdf]

Supporting Information I

Advancing the reproducibility and repeatability of capillary zone electrophoresis-mass spectrometry-based top-down proteomics by an improved capillary coating procedure

Yifan Yue, Fei Fang, Guangyao Gao, Seyed Amirhossein Sadeghi, Jorge A. Colón Rosado, Reyhane Tabatabaeian Nimavard, Mehrdad Falamarzi Askarani, Lance Thorp, Maryam Rahimzadeh Dashtaki, Guijie Zhu*, Liangliang Sun*

Department of Chemistry, Michigan State University, East Lansing, Michigan 48824, United States

* Corresponding authors

Guijie Zhu, email: zhuguiji@msu.edu

Liangliang Sun, email: lsun@chemistry.msu.edu

Table of Contents

	Page	Captions
Table S1	S3	Signal-to-noise ratio and relative standard deviation for peaks P1 and P2 under different capillary and stirring conditions.
Table S2	S4	Migration time, the number of theoretical plates, and separation resolution of Peaks 1 and 2.
Table S3	S5	Summary of migration time and separation resolution of the three capillaries made under the same degassing conditions.
Figure S1	S6	Electropherograms and mass spectra of the standard protein mixture obtained using LPA-coated capillaries prepared with different degassing procedures.
Figure S2	S7	Comparison of peak separation and signal-to-noise ratios for two proteins using LPA-coated capillaries prepared under different degassing conditions.
Figure S3	S8	Migration time and intensity boxplots of two proteins from the *E. coli* sample obtained using LPA-coated capillaries prepared by two individuals under the same degassing condition.
Figure S4	S9	Electropherograms of a standard protein mixture obtained using capillaries prepared by five individuals during the 2025 CE–MS summer school.
Figure S5	S10	Electropherograms of *E. coli* cell lysate obtained using an LPA-coated capillary prepared by 2025 CE–MS summer school participants following the optimized degassing procedure.
Figure S6	S11	Electropherograms of HeLa cell lysate obtained using an LPA-coated capillary prepared under the 75_unstirred condition.

Table S1. Signal-to-noise ratio (SNR) and relative standard deviation (RSD %) for peaks P1 and P2 under different capillary and stirring conditions. Student's t-test was conducted to compare the 75_unstirred condition with the other conditions, and significance levels are indicated by asterisks (the same as Table 1).

Capillary	SNR(P1) Mean/RSD	SNR(P2) Mean/RSD
50_stirred	139/42.6% ***	33.8/56.3% ns
50_unstirred	351/46.8% ***	175/21.3% ***
75_stirred	541/27.8% ns	209/37.2% ns
75_unstirred	471/65.5%	208/27.2%

Table S2. Migration time (MT, min), the number of theoretical plates (N), and separation resolution (R) of Peaks 1 (P1) and 2 (P2). Student's t-test was conducted to compare the 75_unstirred condition with the other conditions, and significance levels are indicated by asterisks (the same as Table 1).

Capillary	MT (P1)<br>Mean/RSD	MT (P2)<br>Mean/RSD	N (P2)<br>Mean/RSD	R (P1 and P2)<br>Mean/RSD
50_stirred	23.2/1.4%	23.7/1.4%	64570/16% ns	1.28/18.24% ***
50_unstirred	25.5/6.3%	26.1/6.4%	123617/54% ns	1.43/18.86% ns
75_stirred	24.9/1.4%	25.4/1.6%	66454/55% ns	1.19/40.67% **
75_unstirred	25.6/1.5%	26.2/1.4%	255853/237%	1.59/18.47%

Table S3. Summary of migration time (MT) and separation resolution of the three capillaries made under the same degassing conditions. 20 CZE-MS runs were performed for each LPA-coated capillary. Significance levels are indicated by asterisks (the same as Table 1, compared to 75_unstirred_1).

Capillary	MT (P1) Mean/RSD	MT (P2) Mean/RSD	Resolution Mean/RSD
75_unstirred_1	25.6/1.5%	26.2/1.4%	1.6/18.5%
75_unstirred_2	21.3/1.7%	21.8/1.7%	1.9/21.0% *
75_unstirred_ZHU	22.6/2.3%	23.0/2.3%	1.6/13.8% ns

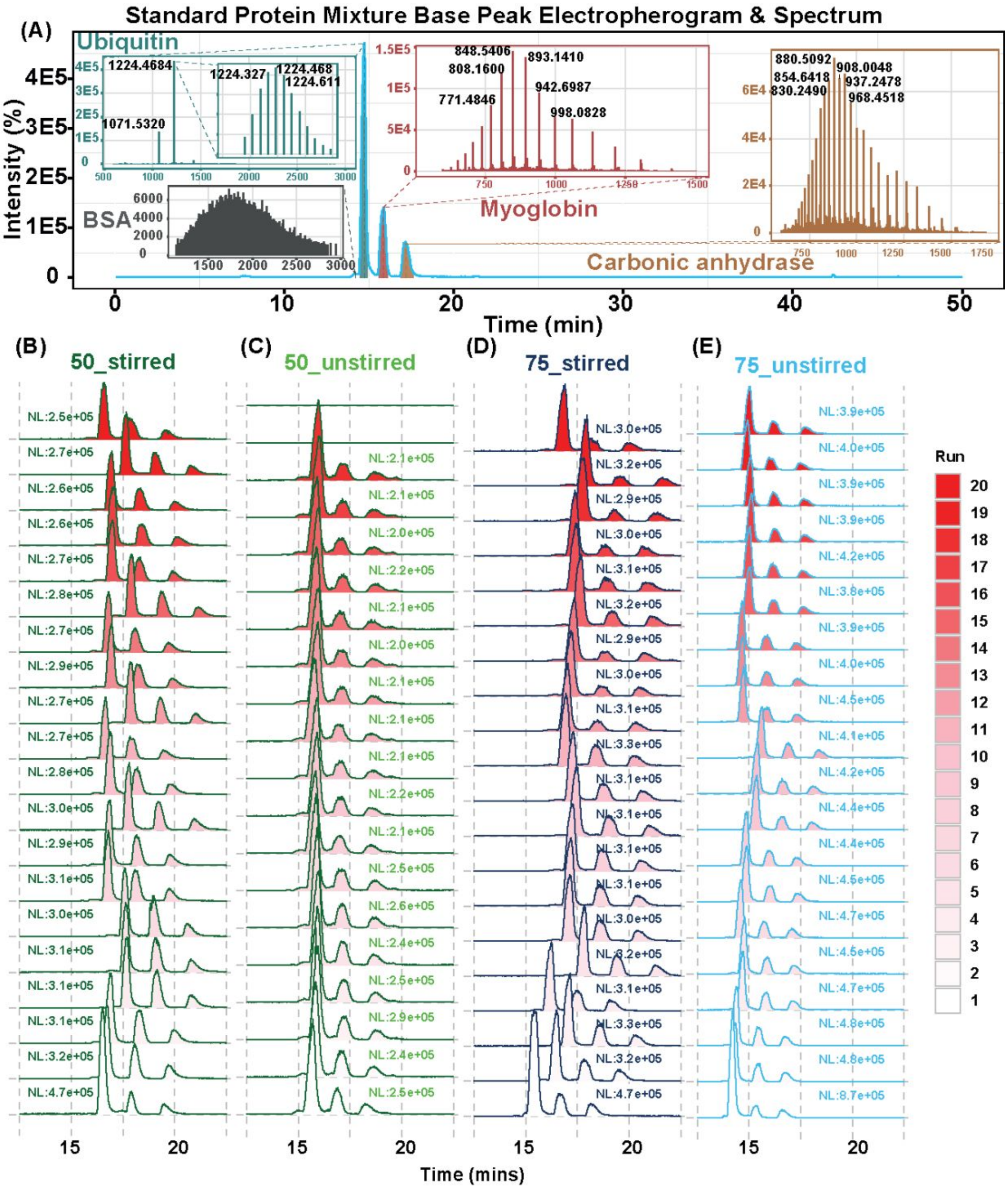

Figure S1. (A) An example electropherogram and mass spectra of the standard protein mixture from the Run 4 using the 75_unstirred LPA-coated capillary. (B), (C), (D), (E) Electropherograms of the standard protein mixture after CZE-MS analysis using LPA-coated capillaries prepared with the four procedures in degassing. NL means normalized intensity level for each run.

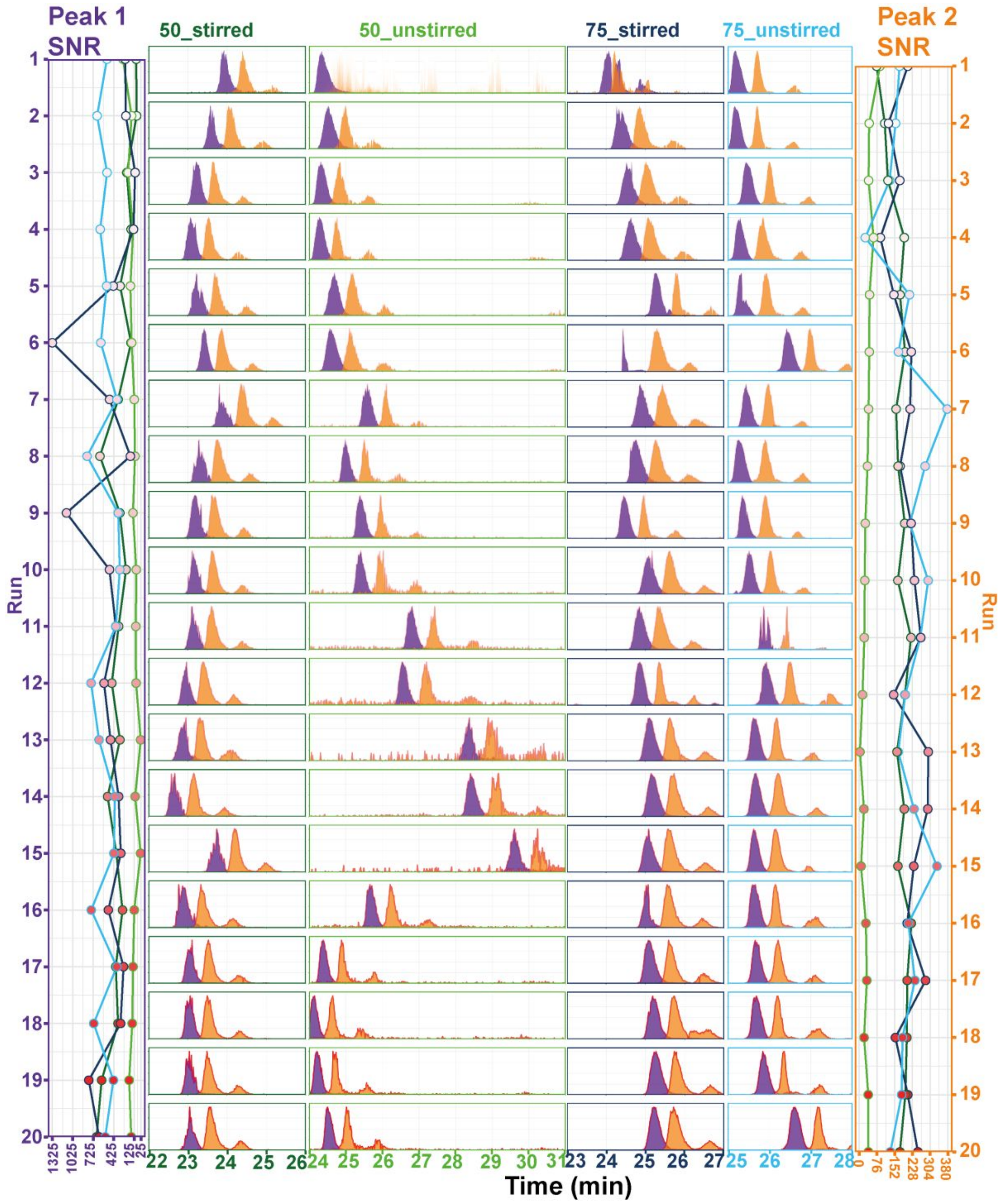

Figure S2. The separation between Peak 1 (~9.8 kDa, purple) and Peak 2 (~12 kDa, orange) and their signal-to-noise ratios (SNRs) were compared across four capillaries prepared under different degassing conditions, each tested over 20 runs. Extracted ion electropherograms (EIEs) were obtained at m/z 1083.03666 ± 0.05 (purple) and m/z 1331.49046 ± 0.05 (orange).

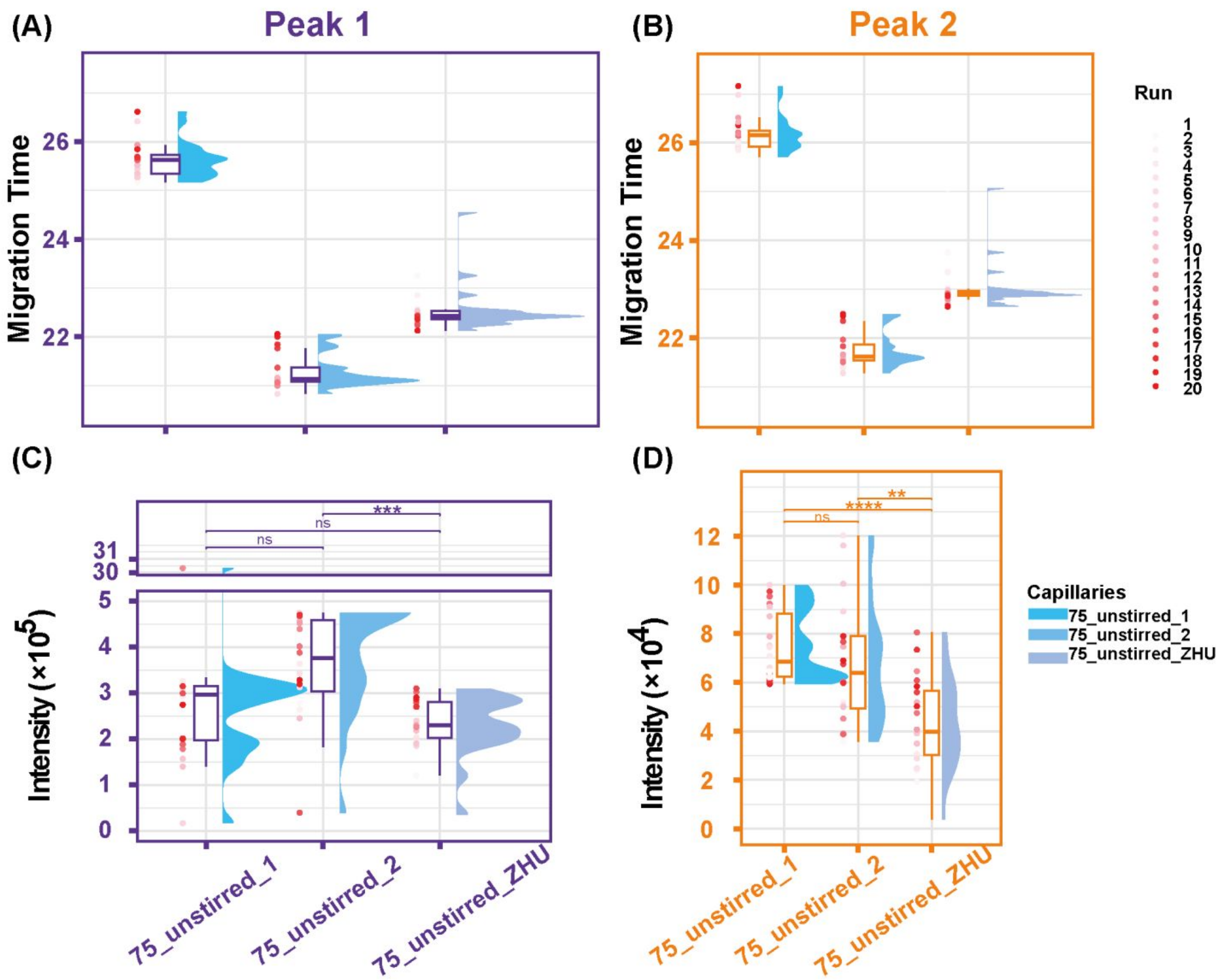

Figure S3. (A, B) Migration time and (C, D) intensity boxplots of the E. coli sample for Peak 1 (purple peak in Fig. 3, ~9.8 kDa) and Peak 2 (orange peak in Fig. 3, ~12 kDa), obtained using three LPA-coated capillaries prepared under the same degassing condition by two individuals. One person made 75_unstirred_1 and 75_unstirred_2, and the other person made 75_unstirred_ZHU.

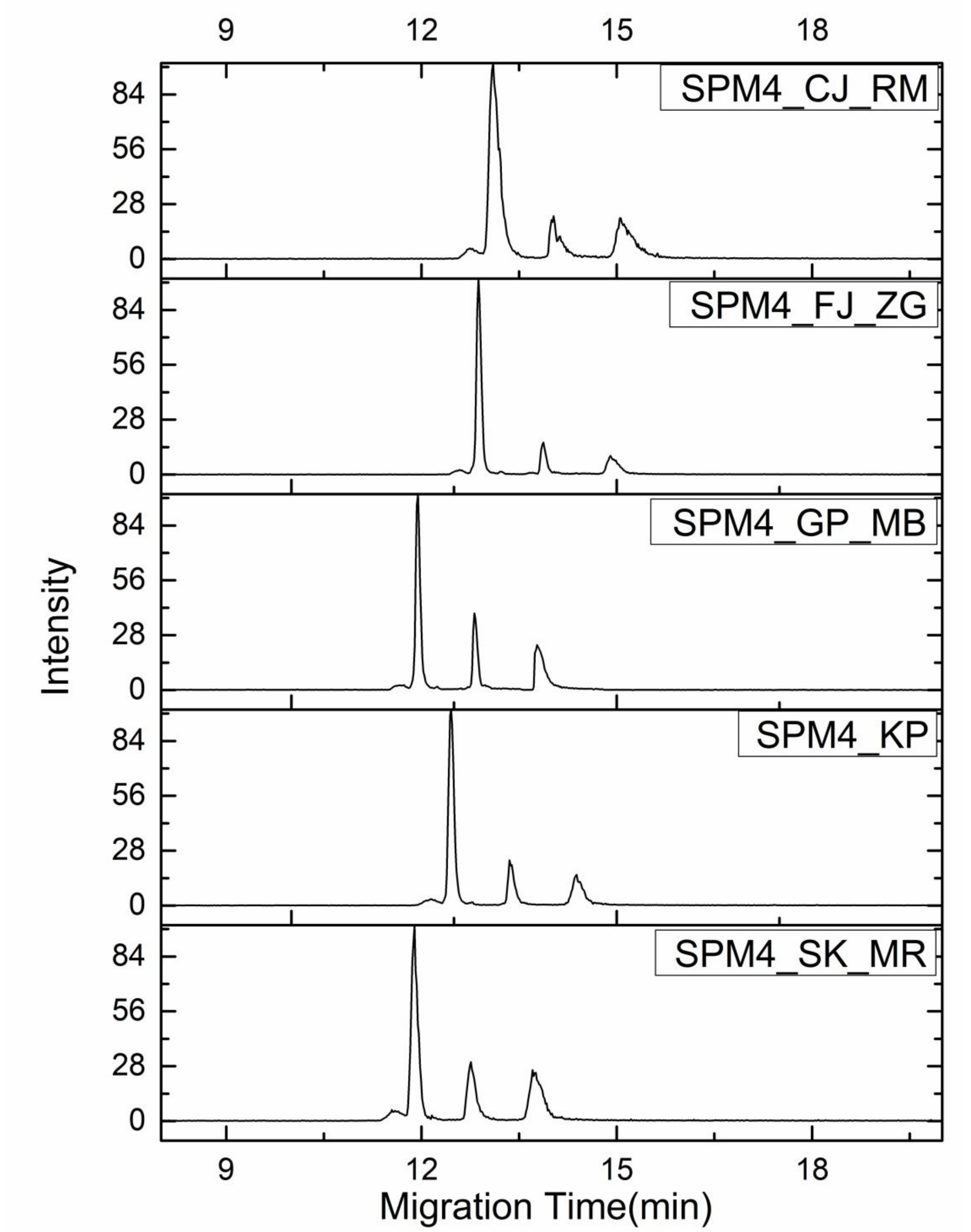

Figure S4. Electropherograms of a standard protein mixture obtained using five capillaries (Capillary_1, 2, 3, 4, and 5) prepared by five different individuals during the 2025 CE-MS summer school, under the 75_unstirred condition.

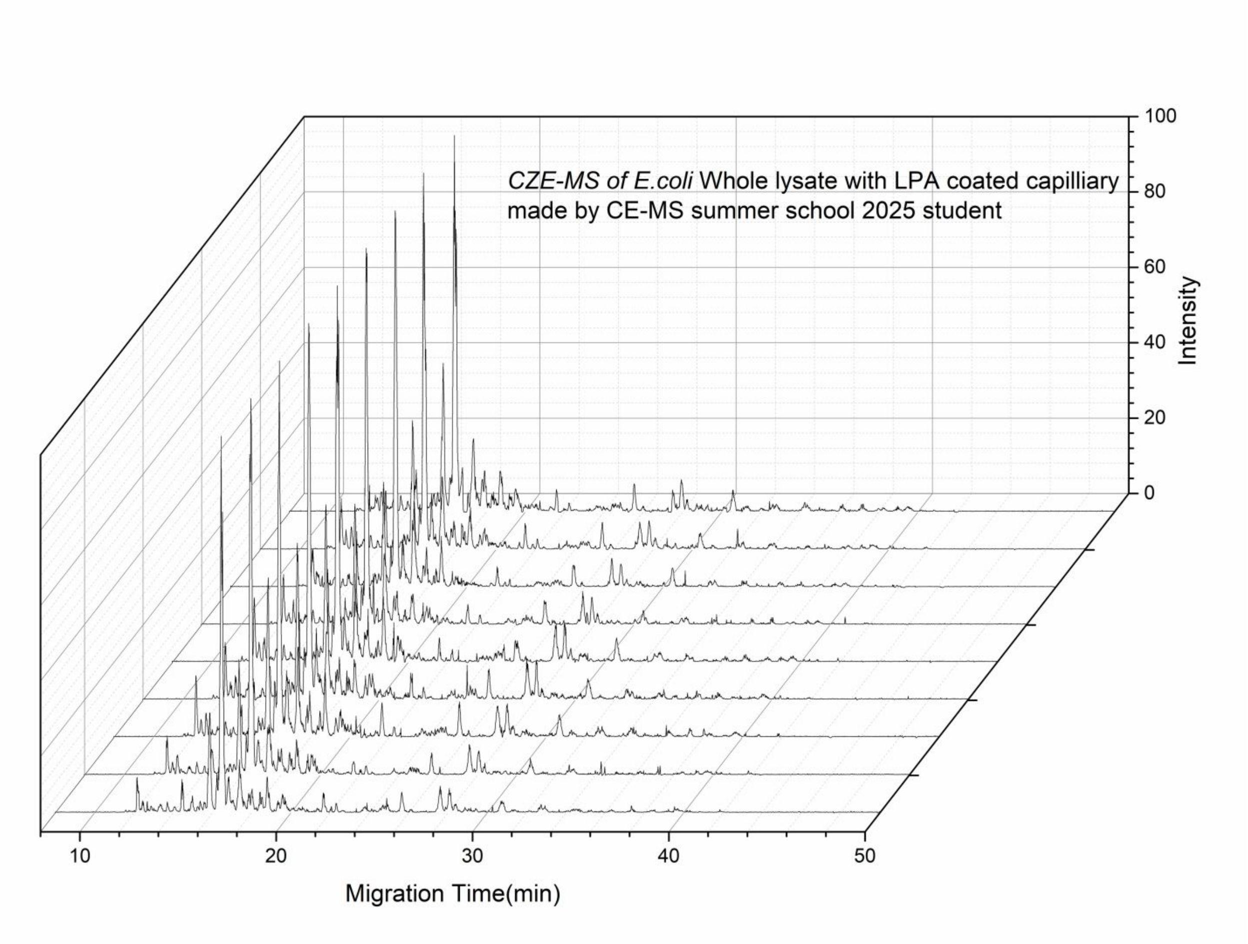

Figure S5. Electropherograms of the *E. coli* cell lysate obtained using one LPA-coated capillary prepared by the 2025 CE-MS summer school participants following the optimized degassing procedure (i.e., the 75_unstirred condition). Nine successive CZE-MS runs of the E. coli sample were performed during one overnight analysis.

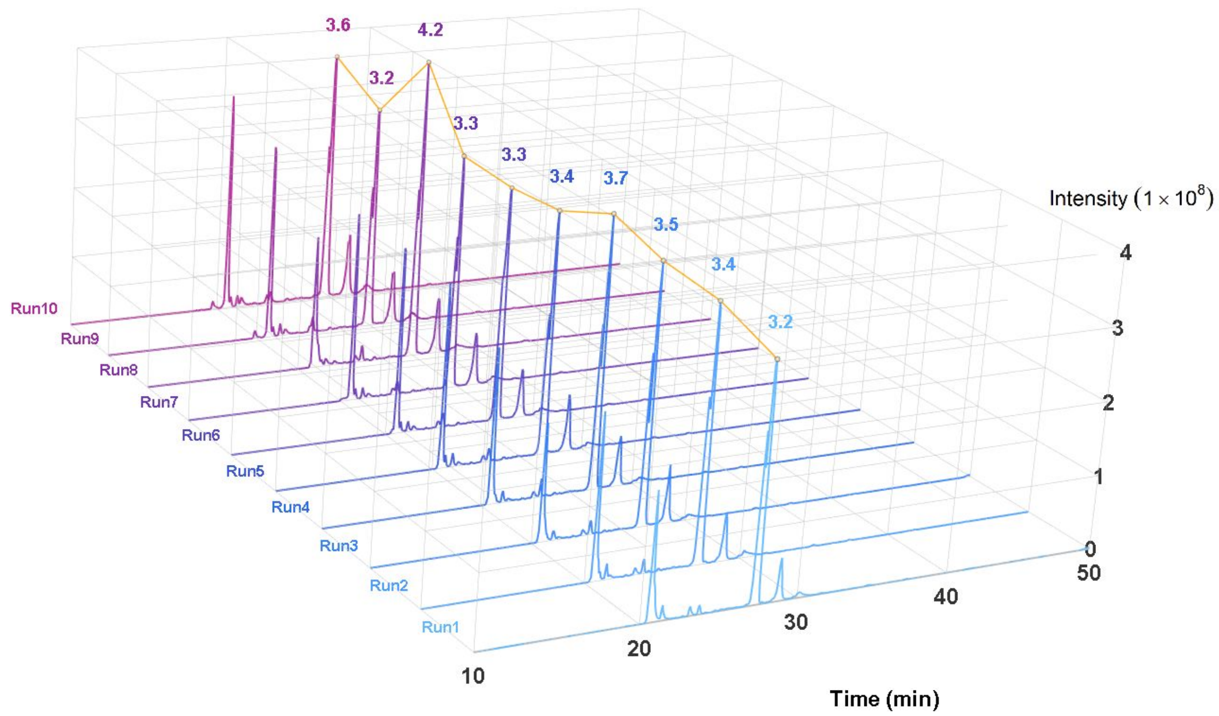

Figure S6. Electropherograms of the Hela cell lysate obtained using the LPA-coated capillary made under 75_unstirred condition. Ten successive CZE-MS runs of the Hela sample were performed.
